# Comparison of Endoscopic and Intraoperative Approaches in the Management of Delayed Gastric Conduit Emptying After Minimally Invasive Esophagectomy: A Single-Center Retrospective Analysis

**DOI:** 10.3390/jcm15082829

**Published:** 2026-04-08

**Authors:** Ramin Raul Ossami Saidy, Philippa Seika, Max M. Maurer, Paul Viktor Ritschl, Matthias Biebl, Dino Kröll, Johann Pratschke, Christian Denecke

**Affiliations:** 1 Chirurgische Klinik, Charité Universitätsmedizin Berlin, Augustenburger Platz 1, 13353 Berlin, Germanymax-magnus.maurer@charite.de (M.M.M.); paul.ritschl@charite.de (P.V.R.); johann.pratschke@charite.de (J.P.);; 2 Berlin Institute of Health, Charité Universitätsmedizin Berlin, 10117 Berlin, Germany; 3 Abteilung für Chirurgie, Ordensklinikum Linz, 4010 Linz, Austria; matthias.biebl@ordenskllnlkum.at; 4 Department of Visceral Surgery and Medicine, Inselspital Bern, Bern University Hospital, University of Bern, 3010 Bern, Switzerland; dino.kroell@insel.ch

**Keywords:** esophageal cancer, delayed gastric conduit emptying, pyloric drainage, pyloric dilatation, botulinum toxin

## Abstract

**Introduction:** As multimodal therapy for esophageal cancer advances, addressing immediate and long-term functional outcomes following surgery has become more important. Despite surgical advancements, delayed gastric conduit emptying (DGCE) remains a primary cause of functional impairment after esophageal cancer resection. The literature addressing pylorus management following minimally invasive esophagectomy (MIE) is scarce. The effects of pyloric drainage with pyloromyotomy or postoperative approaches such as intrapyloric Botox injection or dilatation on the incidence and course of DGCE were the focus of this study. **Methods:** A retrospective analysis of consecutive patients after minimally invasive esophagectomy with thoracic esophagogastric anastomosis and gastric tube reconstruction between 2014 and 2023 was performed. Univariate analyses were used to identify significant patient-, tumor-, and procedure-related factors affecting DGCE. **Results:** A total of 276 patients were included. DGCE was observed in 80 (28.9%) patients. Demographics did not differ with statistical significance. Postoperative complications were not increased in patients with DGCE. Pyloric intervention (PI) did not reduce postoperative occurrence of DGCE (PI: *n* = 19/23.75% compared to no PI: *n* = 62 (30.5%), *p* = 0.342). Median length of hospital stay was significantly longer, and total costs were significantly higher in patients with DGCE (*p* = 0.03 and *p* = 0.047, respectively). Analysis of endoscopic approaches was not associated with a statistically significant difference between botulinum toxin injection and pyloric dilatation with regard to reinterventions. **Conclusions:** While DGCE is frequent after esophagectomy, it is not associated with short-term morbidity but with prolonged total hospital stay and increased costs. Intraoperative pyloric intervention does not influence the incidence of DGCE after esophagectomy and endoscopic management was associated with therapeutic success, but choice of specific, optimal approach remains elusive. Novel concepts, including preoperative dilatation should be investigated.

## 1. Introduction

Improvements in multimodal therapy in patients with esophageal cancer in the long-term put additional focus on functional short- and long-term outcomes related to surgery.

As surgical technology has advanced, the number of patients opting for minimally invasive esophagectomy (MIE) procedures has increased, due to the shorter recovery time and increased quality of life after surgery. Compared to open surgery, MIE has been linked to superior perioperative outcomes such as lower postoperative blood loss and fewer complications, while long-term outcomes, including quality of life, may not differ between approaches [1,2,3].

Certain morbidities are inherent to esophagectomy regardless of the operating method, notwithstanding the advantages of MIE. Vagal nerve resection, commonly required to achieve an adequate oncologic resection, may result in conduit dysmotility and pyloric dysfunction, with subsequent delayed gastric emptying (DGCE) reported in 4 to 50% of patients [4,5,6]. The resulting stasis within the conduit may result in symptoms (i.e., nausea, vomiting, and early satiety) that complicate eating, and increase the risk of aspiration pneumonia and anastomotic stricture, while the impact on anastomotic leakage is disputed [7,8,9,10,11].

Optimal pyloric management after MIE remains unclear and is a matter of ongoing debate [12]. Some surgeons argue against intervention because DGCE may affect only a minority of patients, and nerve regeneration gradually allows recovery of gastric function. In addition, drainage has been linked to complications like increased gastric dumping, esophagitis, and biliary reflux [13,14].

The traditional gold standard for pyloric intervention (PI) has been surgical drainage through pyloromyotomy or pyloroplasty; however, these methods provide more technical challenges and longer recovery times in the modern, less invasive surgical context [15,16,17]. Intrapyloric botulinum toxin injection, a less invasive and easier method of drainage in MIE, has gained popularity since its introduction in 2007 [18,19]. The temporary nature of this therapy has also been cited as an advantage; although improved gastric emptying may be helpful in the immediate postoperative period, its effects diminish as gastric function recovers, reducing the potential for negative long-term effects of drainage [20]. Further, endoscopic pyloric dilatation is a safe and effective approach [8,21,22]. The optimal approach remains unclear, and various combinations of treatment options have been discussed [23,24,25]. While surgical drainage has been reported to be more effective than botulinum toxin injection, some consider it a second-line option, favoring dilatation [6,26].

The effects of intraoperative pyloric drainage on the incidence and risk factors of DGCE, compared to the postoperative use of intrapyloric botox injection and endoscopic dilatation in minimally invasive esophagectomy, were the focus of this study.

## 2. Methods

### 2.1. Study Design

After approval was obtained from the institutional review board, consecutive patients who underwent minimally invasive esophagectomy (MIE) for esophageal malignancy between January 2014 and December 2023 were identified from a prospectively maintained institutional database. The primary study outcome parameter was the occurrence and treatment of DGCE after minimally invasive Ivor–Lewis resection.

### 2.2. Patients

We conducted a retrospective study of all consecutive patients who underwent minimally invasive esophageal resection for cancer of the intrathoracic esophagus or gastroesophageal junction (Siewert type I and type II) between 2015 and 2023 at the Department of Surgery, Charité University Medicine Berlin. The following patients were excluded from the analysis: patients who underwent open resection, open abdominal resection, intraoperative conversion from laparoscopic to open abdominal procedures, non-curatively intended or emergency resection, reconstruction other than a gastric pull-up, or no reconstruction at all. Also, patients who underwent a two-stage reconstruction were excluded.

### 2.3. Outcome Measures and Definitions

The following data were collected from our institutionally prospectively maintained electronic database and electronic patient charts: patient demographics and related comorbidities, tumor-specific variables, use of neoadjuvant treatment, and perioperative and postoperative data. Total costs of hospital stay were retrieved from the institution’s central control and were available for cases from 2017 until now. Tumor staging was conducted using the Union internationale contre le cancer (UICC) staging [27].

Patients were stratified according to pyloric intervention into two groups: no pyloric intervention and pyloric intervention. The PI group comprises patients receiving pyloroplasty (PP) and pyloromyotomy (PM). Stratification was conducted on the treatment of DGCE: first endoscopic approach using botulinum toxin injection, dilatation, or combination. Further, stratification accounted for multiple interventions and treatment switches during the patient’s course.

Diagnosis of DGCE was initiated upon clinical suspicion (high reflux via nasogastric tube beyond 3rd day after surgery, nausea, vomiting, or persistent feeling of fullness despite only small meals) and was based on these findings, confirmed either radiologically using contrast agent or with endoscopic findings. Not all patients received radiological confirmation of DGCE before endoscopic verification.

DGCE was divided into early (<14 days after surgery) and late (>14 days after surgery) [28]. Only patients with DGCE class 1 were included [6].

Endoscopic treatment approaches were chosen based on the treating clinicians’ expertise. Similarly, a change in treatment from botulinum injections to dilatation and vice versa was decided upon the individual patient’s course and endoscopists’ judgment.

To ensure comparability in regard to time period or operator, the observational period was divided into the years 2015–2019 and afterwards, and specific analysis for individual surgeons was conducted.

### 2.4. Surgical Techniques

The patients underwent a standard Ivor–Lewis esophagectomy with a two-field lymphadenectomy and a minimally invasive intrathoracic circular stapled anastomosis via a transabdominal (laparoscopic or robotic-assisted) and right thoracal (thoracoscopic or robotic-assisted) approach. A 25 mm- or 29 mm-diameter 2-row circular stapler was used for anastomoses.

The abdominal part comprised a standard D2-lymphadenectomy around the branches of the celiac trunk, gastric cardia, and lower mediastinum, and gastrolysis with careful preservation of the right gastroomental pedicle utilizing a partial Kocher maneuver.

Under preservation of the right gastric vessels, a partial division of the stomach from the lesser curvature starting approximately 6 cm proximal to the pyloric ring was performed to create a 5- to 6 cm-wide gastric tube, which was later completed during the thoracic phase through the retrieval incision using a laparoscopic linear stapler with green and blue cartridges. After careful dissection of the anteriorly widened esophageal hiatus, the abdominal phase was completed.

Next, the patient was repositioned in a left lateral position to perform thoracic dissection. After achieving stable left-sided single-lung ventilation, the thorax was entered through a 6 cm mini-thoracotomy at the level of the posterior axillary line in the fourth intercostal space, and an Alexis foil self-retaining ring (Applied Medical, Rancho Santa Margarita, CA, USA) was inserted. A 12 mm trocar was introduced in the 6th intercostal space (ICS), and two 12 mm trocars were introduced in the 9th ICS. For robotic access, the trocars were positioned at 4, 6, 8, and 10. ICS along the posterior axillary line, together with one 12 mm assist trocar in the 7. ICS. The inferior pulmonary ligament was divided, and the esophagus was mobilized to visualize the mes, which was then transsected close to the aorta. Next, the esophagus was looped with a silicone drain for retraction and circular dissection. The azygous vein was routinely divided with an endoscopic stapler or secured by two locking clips on either side and transected. Mediastinal compartment lymphadenectomy along the esophagus, carinal region, and around the azygos vein was routinely performed. The intended transection level of the esophagus was confirmed by intraoperative endoscopy, and the esophagus was divided with a linear endoscopic stapler. The gastric tube was brought into the thorax, and the specimen was retrieved through the minithoracotomy incision. Outside the thorax, the gastric tube was completed, and the specimen was removed. After gross back table inspection, frozen section examination of the proximal margin was performed.

### 2.5. Techniques of Pyloric Intervention

The decision of whether and which type of pyloric intervention to use was dependent upon the surgeon’s preference. Pyloric drainage included intraoperative endoscopic pyloromyotomy or surgical pyloroplasty.

Endoscopic dilatation was performed by pneumatic balloon dilatation, using a 30 mm balloon. Botulinum toxin injection was performed by applying 25 IE into each quadrant of the pyloric sphincter in a single session.

### 2.6. Postoperative Care and Follow-Up

Nasogastric tubes were given to all patients after surgery. Routine radiologic studies for delayed gastric emptying (DGCE) were not conducted. The advancement of the diet was based on clinical presentation. High output of nasogastric tube drainage, defined as exceeding 500 mL of intake, was considered an indicator of DGCE.

Follow-up was conducted by routine visits at our department’s outpatient clinical and reports from our clinical information system, as well as routine follow-up of our cancer center.

### 2.7. Statistical Analysis

By its retrospective character, the study design was exploratory. Quantitative and qualitative variables were expressed as medians with interquartile range and frequency. For the testing of statistically significant differences, cross-tables with Chi-squared or Fisher’s exact test were used for nominal-scaled variables. The *t*-test was applied for continuous, normally distributed variables. For the testing of nonnormally distributed values, the Mann–Whitney U-test or Kruskal–Wallis test was chosen. In case of missing data, only complete cases were analyzed for the specific analysis. *p*-values < 0.05 were considered statistically significant. Statistical analysis was performed using the SPSS software package (Version 29.0).

## 3. Results

In total, 276 patients undergoing minimally invasive esophagectomy with gastric conduit reconstruction were included in this study. DGCE was diagnosed in 80 (29.0%) patients. Radiological confirmation before endoscopy was achieved in 37 (46.3%). Early DGCE occurred in 46 (16.7%) patients. Incidence of DGCE did not differ between observational time periods (2015–2019: *n* = 31/27.7% vs 2020–2023: *n* = 49/29.9%, *p* = 0.69). Median follow-up was 9.0 (16.5) months. The baseline demographics and clinical characteristics are shown in Table 1. No statistically significant differences between the group of patients developing DGCE and those who did not were found when analyzing oncological parameters such as UICC stage, neoadjuvant therapy, or patient characteristics, e.g., age, sex, body mass index (BMI), or comorbidities. During the observational period, four main operators were identified, and the occurrence of DGCE or main complications did not differ between them (*p* = 0.67 and *p* = 0.70, respectively). Median time to first intervention for DGCE was 12 (45) days.

Comparing the impact of laparoscopic and robotic-assisted surgery on DGCE, overall incidence did not differ (*n* = 54/26.3% vs. *n* = 26/36.6%, *p* = 0.1). Also, no difference was found in the incidence of early vs late DGCE or time to first intervention.

While the rate of overall postoperative complications as well as specific complications did not occur more frequently in patients with DGCE, overall length of stay was significantly prolonged, and total costs of hospital stay were significantly increased in these patients (*p* = 0.030 and *p* = 0.047, respectively), for details see Table 2.

No complications from PI were recorded. Intraoperative pyloric intervention showed no effect on the occurrence of DGCE, as out of 78 patients undergoing the procedure, 19 (24.4%) developed DGCE, compared to 61 (30.8%) out of 198 who did not (*p* = 0.288). PI was significantly associated with the main operator, as one surgeon performed *n* = 64 (82.1%) of all procedures (*p* < 0.001). Median time to first intervention was 10 (29.0) days in patients with pyloric intervention and 18 (26.3) days in those without, but did not reach statistical significance (*p* = 0.406).

Most patients received an injection of botulinum toxin as the first treatment strategy for DGCE (*n* = 66/82.5). Multiple interventions were necessary in 35 (43.8%) patients, and a treatment switch (dilatation following initial botulinum toxin and vice versa) was conducted in 25 (31.3%) patients. No perforation during endoscopy was documented, but three (1.1%) patients were found with endoscopy-associated aspiration pneumonia. There were no statistically significant differences between the groups of early and late DGCE in endoscopic treatment approaches, see also Table 3.

Multiple endoscopic interventions for DGCE were necessary in 35 (43.8%) patients. Intraoperative pyloric intervention showed no impact on avoidance of multiple interventions or on total number of endoscopies (*p* = 0.251 and *p* = 0.593, respectively).

Further, 27 (40.9) patients undergoing botulinum injection needed multiple interventions, compared to 5 (55.6%) with initial dilatation treatment and 3 (60.0%) patients that received initial treatment combination, resulting in no statistically significant difference (*p* = 0.532).

Comparison of initial treatment using botulinum toxin injection or pyloric dilatation showed no statistically significant difference in the necessity of eventual treatment switch (*p* = 0.199).

While 53 (70.7%) out of 75 patients who eventually received botulinum injection only received one injection intervention, 12 (16.0%) needed a second intervention, and 10 (13.3%) patients received more than two.

31 patients received pyloric dilatation, with one session being sufficient in 22 (71.0%), but 6 (19.4%) required a second dilatation, and two (6.5%) patients more than two.

Subgroup analysis did not reveal a significant impact of intraoperative pyloric intervention for patients with high thoracic tumor manifestation (25 cm from the dental arch) on the occurrence of DGCE. A statistically significant reduction in the number of interventions was observed for patients with tumors below 25 cm from the dental arch that did not undergo pyloric intervention (*p* = 0.048). Similarly, subgroup analysis of patients with dilatation vs botulinum toxin as first intervention and high tumor manifestation did not reveal statistically significant differences in required interventions for therapy of DGCE (*p* = 0.472). No patient was documented as undergoing surgical revision.

## 4. Discussion

Delayed gastric conduit emptying after esophagectomy occurs frequently after esophagectomy, with a pooled incidence of 15.9% in a recent meta-analysis, but its prevalence might be higher, even after 2 years [29,30]. Its incidence might be reduced by a minimally invasive approach, but data are scarce [31]. The rate of DGCE undergoing sole minimally invasive procedures observed in this study of 29.0% corresponds with published data and did not differ in regard to the observational period. We found no difference in the incidence of DGCE between laparoscopic and robotic esophagectomy. Of note, we did not find any data comparing the incidence of DGCE regarding these approaches.

DGCE was associated with increased postoperative length of stay, as it usually requires prolonged gastric tube decompression and hampers early food intake. These aspects are also identified as key factors in the perioperative rehabilitation after esophagectomy by the Enhanced Recovery After Surgery (ERAS) society, highlighting the importance of effective management of DGCE [32].

Unsurprisingly, total costs in the group of patients with DGCE were significantly increased, certainly partially reflecting the need for (re-)interventions and prolonged hospital stay.

The term intraoperative pyloric intervention may comprise different approaches, including surgical techniques such as pyloroplasty, pyloromyotomy, but also endoscopic interventions, including botulinum toxin injection, pyloric dilatation, and even combinations, and has been linked to reduced incidence of DGCE [17]. In this study, only pyloroplasty (PP) and pyloromyotomy (PM) were classified as PI, and no reduction in postoperative DGCE was observed in these patients. The PI in this study was mainly performed by one surgeon, assuring a homogeneous outcome without operational differences. Studies reporting on surgical PI for open esophagectomy have reported a reduction in early DGCE without effect on major complications such as anastomotic leakage or oncological outcome [14,16,33]. Interestingly, a meta-analysis of randomized controlled trials concluded that PI was not necessary, and even the results on DGCE were inconclusive [34]. Consequently, ERAS recommendations do not endorse pyloric drainage, and our findings support these notions, especially in the context of MIE [32].

The optimal treatment strategy for DGCE remains unclear. Benefits and disadvantages in terms of patient safety or costs have to be evaluated, as both prophylactic treatment and active postoperative surveillance remain strategies. Francken et al. investigated costs for delayed gastric emptying after pancreatoduodenectomy and found significantly higher costs for these patients, despite the absence of other complications [35].

While generally, endoscopic approaches have been well-established due to low invasiveness and good outcomes, a recommendation for a specific initial intervention method does not exist [29]. Botulinum toxin injection and dilatation have been reported with comparable success rates, but with frequent need for reintervention until complete therapeutic success. In this study, the outcome of both interventions was comparable, and reinterventions as well as the switch of endoscopic management strategy were similar. The initial combination of both interventions has been linked to favorable outcomes [23,36]. The number of patients receiving initial combination treatment was low, with only five patients, and, therefore, statistical meaningfulness is limited. While the treatment option of Gastric Peroral Endoscopic Myotomy (G-POEM) is available at our clinic, in this study, we did not assess patients undergoing this approach.

Preoperative pyloric intervention has been discussed in the management of DGCE, and results are promising; protocols for randomized controlled studies have been published [37,38].

This study is certainly limited by its retrospective nature as well as its possible selection/indication bias towards treatment in, e.g., endoscopic approaches. Further, standardized grading of symptoms or symptom relief and definition of therapeutic success after interventions were not conducted. Data on the necessity for prolonged parenteral nutrition and patient-oriented parameters, including weight loss and (laboratory) nutritional status, were not available in this study. Routine follow-up for all patients in regard of DGCE was not performed, and thus, relapse/therapy-refractory cases with a switch in treating institutions might not be accounted for. Consequently, underestimation of DGCE and therapeutic failure is possible. Especially for subgroup analysis, low patient numbers might hamper statistical meaningfulness and limit statistical testing approaches and options. Further, statistical observations from sole univariate analysis might underestimate the influence of potential confounders. However, the distinction of treatment approaches and its focus on symptomatic DGCE rather than radiological diagnosis only may support the notion of endoscopic over surgical approaches.

## 5. Conclusions

Delayed gastric conduit emptying remains frequent after minimally invasive esophagectomy. While optimal endoscopic treatment choices remain unclear, in this study, intraoperative pyloric interventions were not associated with a preventive effect. Endoscopic combination treatments, as well as preoperative concepts, should be the focus of research in the era of minimally invasive esophagectomy.

## Figures and Tables

**Table 1:** Clinical characteristics.

Variable	Overall (*n* = 276)	No DGCE (*n* = 196)	DGCE (*n* = 80)	*p* *
Sex, *n* (%)				0.231
Male	200 (72.5)	138 (70.4)	62 (77.5)	
Female	76 (27.5)	58 (29.6)	18 (22.5)	
Median age at resection, years (IQR)	65 (15.0)	64.0 (15.0)	65.0 (16.0)	0.450
Median BMI, kg/m^2^ (IQR)	26.0 (6.0)	25.1 (6.8)	26.0 (5.0)	0.68
Tumor location, *n* (%)				0.148
Esophagus	145 (52.5)			
GEJ	111 (40.2)	105 (59.7	40 (50.0)	
Missing data	20 (7.2)	71 (40.3)	40 (50.0)	
Comorbidities, *n* (%)				
Diabetes mellitus type II	44 (15.9)	35 (17.9)	9 (11.3)	0.169
Arterial hypertension	149 (54.0)	108 (55.4)	41 (51.2)	0.532
Coronary heart disease	41 (14.9)	32 (16.4)	9 (11.3)	0.275
Cardiac arrhythmia	34 (12.3)	20 (10.3)	14 (17.5)	0.097
UICC stage at diagnosis				0.429
I	12 (5.4)			
II	17 (7.7)	8 (5.2)	4 (6.0)	
III	164 (74.2)	9 (5.8)	8 (11.9)	
IV	28 (12.7)	116 (75.3)	48 (71.6)	
Missing data	55 (19.9)	21 (13.6)	7 (10.4)	
Neoadjuvant therapy, *n* (%)				
Chemotherapy	242 (87.7)	176 (90.7)	66 (83.5)	0.090
Radiochemotherapy	120 (43.5)	92 (46.9)	28 (35.0)	0.069
DGCE, *n* (%)				-
Early	46 (16.7)	-	46 (57.5)	
Late	34 (12.3)	-	34 (42.5)	
Technical approach, *n* (%)				0.10
Laparoscopic	205 (74.3)	151 (73.7)	54 (26.3)	
Robotic	71 (25.7)	45 (63.4)	26 (36.6)	

* Comparison between DGCE vs no DGCE. DGCE—delayed gastric conduit emptying, IQR—interquartile range; BMI—body mass index; GEJ—gastroesophageal junction.

**Table 2:** Postoperative outcome and DGCE.

Variable	No DGCE *n* = 196	DGCE (*n* = 80)	*p*
AL, *n* (%)	24 (12.2)	15 (18.8)	0.159
Pulmonary complications, *n* (%)	59 (30.3)	34 (42.5)	0.051
Clavien–Dindo classification			0.121
≤3a	165 (84.2)	61 (76.3)	
≥3b	31 (15.8)	19 (23.8)	
Median ICU, days (IQR)	4.0 (3.0)	4.0 (4.75)	0.550
Median LOS, days (IQR)	15.0 (10.0)	19.0 (26.5)	0.030
Median costs, EUR (IQR)	30,112 (11,658.5)	39,977 (37,355.0)	0.047

DGCE—delayed gastric emptying, AL—anastomotic leak, ICU—intensive care unit, LOS—length of stay, EUR—Euro.

**Table 3:** Treatment of DGCE.

Variable	All Patients with DGCE (*n* = 80)	Early DGCE (*n* = 46)	Late DGCE (*n* = 34)	*p* *
Initial endoscopic treatment				0.060
Botulinum toxin	66 (82.5)	34 (73.9)	32 (94.1)	
Dilatation	9 (11.3)	8 (17.4)	1 (2.9)	
Combination	5 (1.8)	4 (8.7)	1 (2.9)	
Treatment switch	25 (31.3)	17 (37.0)	8 (23.5)	0.20
Multiple interventions	35 (43.8)	21 (45.7)	14 (41.2)	0.69
Botulinum toxin	22	10	12	-
Median interventions (IQR)	1 (0)	1 (0)	1 (1)	0.097
Dilatation	8	8	0	-
Median interventions (IQR)	0 (0)	0 (1)	0 (1)	0.182
Intraoperative pyloric intervention (%)	19 (23.75)	9 (19.6)	10 (29.4)	0.306
Median time to first intervention, days (IQR)	12 (45)	6.5 (5.5)	32.5 (30.3)	0.007

* Comparison between DGCE vs. no DGCE; DGCE—delayed gastric conduit emptying, IQR—interquartile range.

## Data Availability

The datasets presented in this article are not readily available because of data safety concerns of our local institution.

## Abbreviations

BMI	Body mass index
DGCE	Delayed gastric conduit emptying
GEJ	Gastroesophageal junction
G-POEM	Gastric Peroral Endoscopic Myotomy
IQR	Interquartile range
(RA)MIE	(Robotic-assisted) minimally invasive esophagectomy
PI	Pyloric intervention
PM	Pyloromyotomy
PP	Pyloroplasty
UICC	Union internationale contre le cancer
